# *Candida albicans* onychomycosis coexisting with nail scabies: a diagnostic challenge in an elderly woman

**DOI:** 10.1016/j.mmcr.2026.100835

**Published:** 2026-11-06

**Authors:** Jie Zhou, Nan Xu, Min Chen

**Affiliations:** a Department of Dermatology, The Seventh People's Hospital Affiliated to Shanghai University of Traditional Chinese Medicine, Shanghai, China; b Department of Dermatology, Shanghai East Hospital Affiliated to Tongji University, Shanghai, China; c Department of Dermatology, Zhoupu Hospital Affiliated to Shanghai University of Medicine and Health Sciences, Shanghai, China

**Keywords:** Onychomycosis, Nail scabies, Diagnosis

## Abstract

Nail scabies is rare and may closely mimic onychomycosis, particularly in patients without typical cutaneous lesions. Here we report an 82-year-old Chinese woman initially suspected of having onychomycosis, whereas direct microscopic examination of nail scrapings unexpectedly revealed *Sarcoptes scabiei* mites together with fungal hyphae in the same microscopic field. Fungal culture yielded yeast subsequently identified as *Candida albicans* (*C. albicans*) by matrix-assisted laser desorption/ionization time-of-flight mass spectrometry (MALDI-TOF MS). Based on these findings, nail scabies coexisting with *C. albicans* onychomycosis was diagnosed. The patient was successfully treated with topical crotamiton under occlusion combined with bifonazole cream. This case demonstrates that fungal infection and parasitic infestation may coexist within the same nail unit and highlights the importance of combined parasitological and mycological evaluation in patients with atypical nail dystrophy.

## Highlights

• Nail scabies is easily to be misdiagnosed as onychomycosis, especially presenting as isolated nail disease without typical cutaneous manifestations, expanding the clinical spectrum beyond crusted scabies.
• This case demonstrates microbiologically confirmed coexistence of *C. albicans* onychomycosis and nail scabies, emphasizing the importance of combined parasitological and mycological evaluation in patients with atypical nail disorders.

## 1 Introduction

Onychomycosis is a common superficial fungal infection of the nail unit, accounting for approximately 50% of all nail disorders, and is also more prevalent among elderly individuals [1]. Meanwhile, scabies is a contagious infestation of skin and cutaneous appendages caused by *Sarcoptes scabiei* (*S*. *scabiei*) [2]. Although nail involvement in scabies (hereafter referred to as nail scabies) is more frequently observed among patients with crusted scabies [2], it has rarely been reported in patients with ordinary scabies [3]. Notably, elderly individuals are a vulnerable population for both onychomycosis and scabies [4]. Owing to its higher prevalence in clinical practice, onychomycosis can be more readily suspected from clinical manifestations. Moreover, clinical manifestations of onychomycosis and nail scabies within this population may be atypical, resulting in diagnostic challenges [3], [5]. Herein, we report a rare case of *Candida albicans* (*C. albicans*) onychomycosis coexisting with nail scabies. This case highlights the diagnostic challenge of distinguishing between fungal and parasitic nail disorders.

## 2 Case description

An 82-year-old Chinese woman with a 6-year history of chronic renal insufficiency and cerebral infarction presented with a 1-month history of progressive nail dystrophy involving the right hand. According to her family members, several fingernails had gradually become thickened and discolored, without associated pruritus, pain, or other discomfort. Dermatologic examination revealed four dystrophic, thickened, and yellowish nails on the right hand, accompanied by periungual erythema, swelling, and hyperkeratotic scales (Fig. 1A and B). No burrows, vesicles, or other typical cutaneous manifestations of scabies were observed elsewhere. Onychomycosis was initially suspected, and nail scrapings with subungual debris were obtained for direct microscopic examination. Unexpectedly, direct microscopic examination using fungal fluorescence staining solution (Jiangsu Nuoge Biotechnology Co., Ltd.) revealed *S. scabiei* mites and fungal hyphae within the same microscopic field, demonstrating simultaneous parasitic and fungal involvement of the nail unit (Fig. 2A and B). Fungal culture of nail specimens yielded yeast growth, and this yeast was subsequently identified as *C. albicans* by matrix-assisted laser desorption/ionization time-of-flight mass spectrometry (MALDI-TOF MS). The patient was diagnosed with nail scabies concomitant with *C. albicans* onychomycosis. Treatment was initiated with topical crotamiton cream under continuous occlusive dressing combined with bifonazole cream. After two weeks of treatment, significant improvement in nail dystrophy and periungual inflammation was observed. Follow-up direct microscopy revealed no fungal elements or mites. No recurrence was observed during the 3-month follow-up period.

**Fig. 1:**
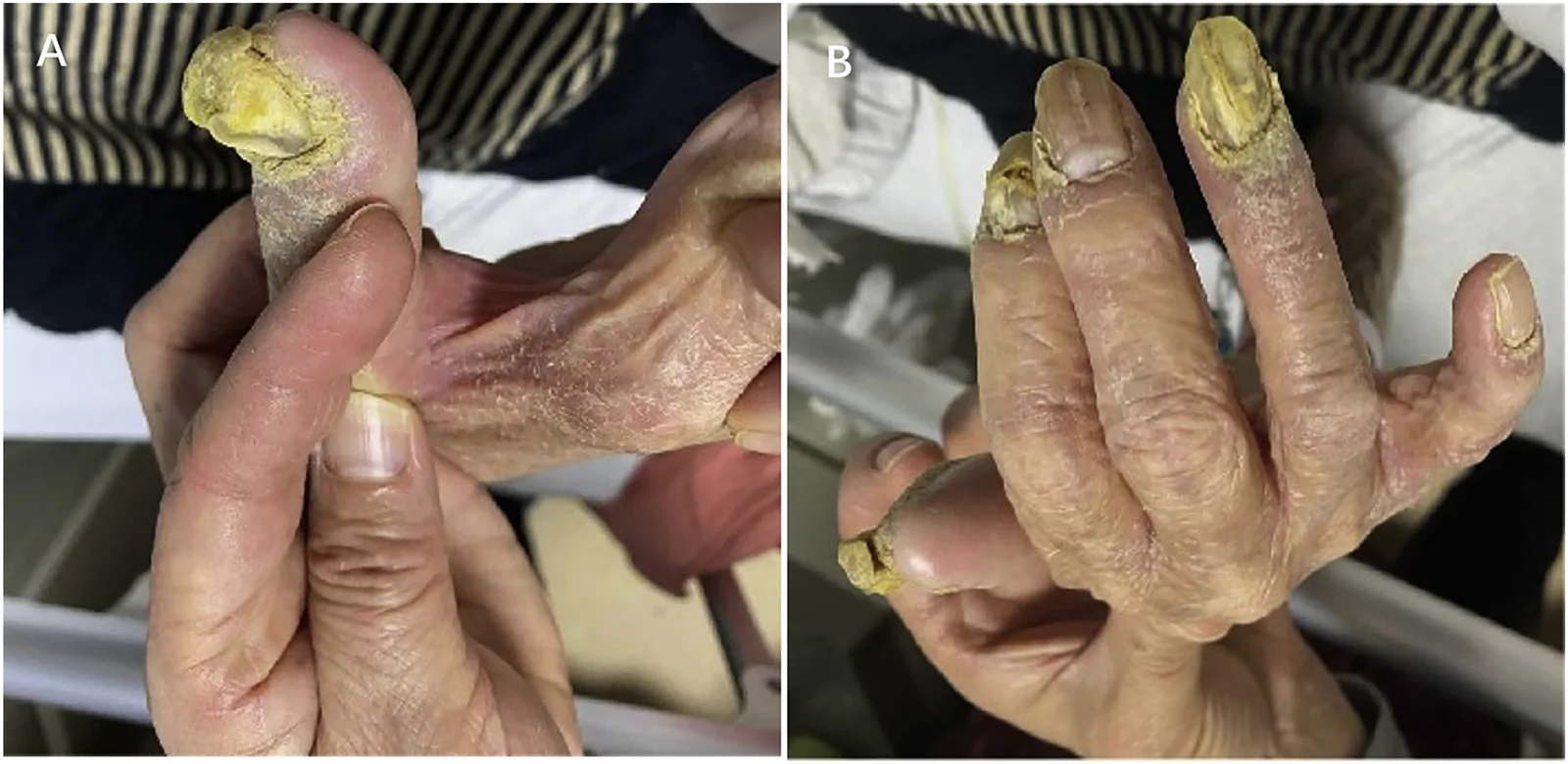
**Clinical manifestations of nail scabies coexisting with *Candida albicans* onychomycosis.** (A, B) Clinical examination revealed multiple dystrophic, thickened, and yellowish fingernails with periungual erythema, swelling, and hyperkeratotic scales involving the right hand. The nail changes clinically mimicked onychomycosis.

**Fig. 2:**
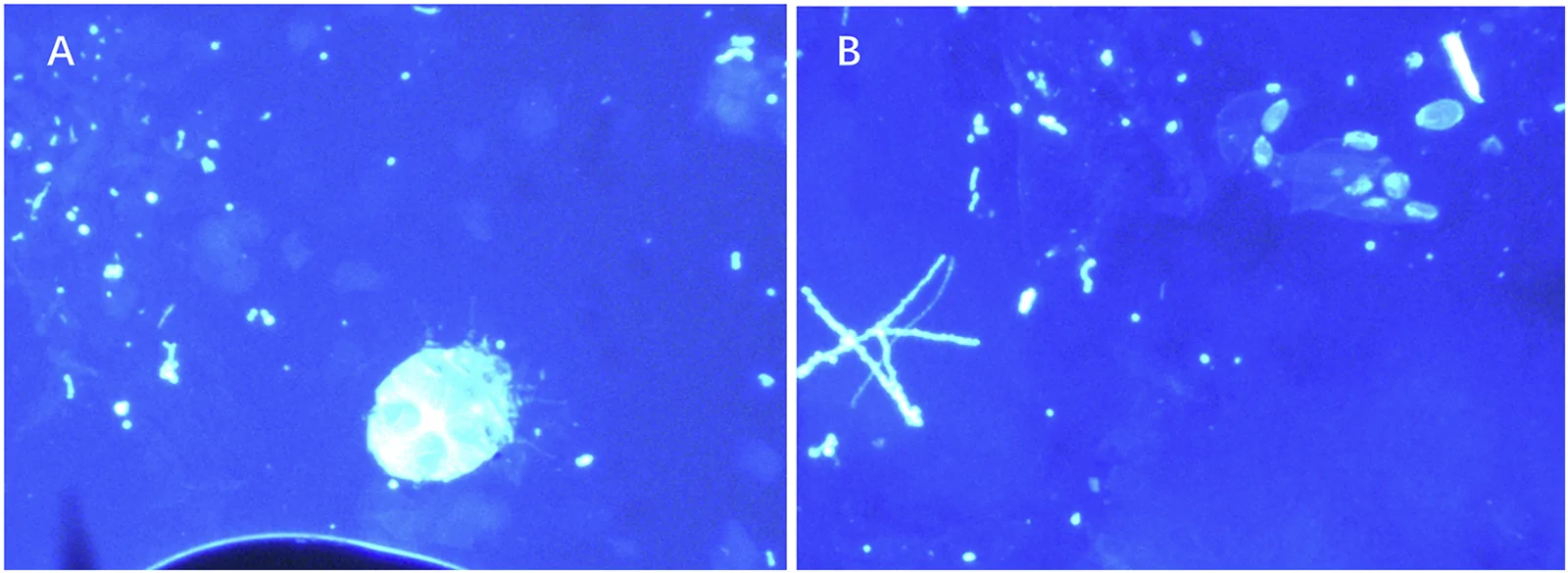
**Simultaneous visualization of parasitic and fungal elements within the nail specimen using fungal fluorescence staining.** (A) A *Sarcoptes scabiei* mite identified by direct microscopy. (B) Fungal hyphae observed in the same microscopic field, demonstrating concurrent parasitic and fungal involvement of the nail unit.

## 3 Discussion

Nail involvement is an uncommon manifestation of scabies and is frequently overlooked because its clinical presentation may mimic other nail disorders, particularly onychomycosis [3]. Previous reports have shown that nail scabies occurs predominantly in patients with crusted scabies, characterized by subungual hyperkeratosis, nail dystrophy, discoloration, and onycholysis [5], [6].

In our review of recently reported cases of nail scabies (Table 1), most affected patients were elderly or had underlying systemic conditions. Several cases occurred in the context of crusted scabies, in which extensive hyperkeratotic lesions and a high mite burden facilitated nail infestation [6]. Nail involvement in crusted scabies was described by De Paoli and Marks in 1987 [7], who emphasized the difficulty of eradicating mites from the nail unit. However, recent reports have expanded the clinical spectrum of nail scabies, showing that classical or ordinary scabies may also present with isolated nail involvement without extensive cutaneous manifestations [3], [8], [9]. Notably, cases of nail-predominant or isolated nail scabies have been reported with increasing frequency; these cases were often initially misdiagnosed as onychomycosis or nail psoriasis because of their overlapping clinical features [3], [10]. For example, Isogai et al. described nail scabies as the initial manifestation of ordinary scabies [8], and Zou et al. [9] reported an atypical presentation of ordinary scabies primarily confined to the nail unit. More recently, Veraldi et al. demonstrated that ordinary scabies may present as isolated nail disease mimicking onychomycosis, even in the absence of ordinary cutaneous lesions [3]. In these cases, direct microscopic examination of nail scrapings is essential for detecting *S*. *scabiei* and establishing diagnosis.

**Table 1:** Clinical characteristics of reported cases of scabies with nail involvement and the present case.

Reference	Time	Age/Sex	Country	Underlying disease	Scabies subtype	Nail manifestations	Cutaneous manifestations	Mycological evaluation	Diagnostic method	Treatment
De Paoli et al., 1987	1987	24/male	USA	Down syndrome	Crusted scabies	Multiple fingernails and toenails with thick subungual debris, onycholysis, dystrophy	Hyperkeratotic plaques on palms and soles; burrows present	Subsequent *Trichophyton rubrum* onychomycosis during follow-up	Direct microscopy of nail scrapings (KOH)	40% urea nail dissolution, partial nail avulsion, and topical lindane
Isogai et al., 2002	2002	86/male	Japan	Not reported	Ordinary scabies	Thickened and whitened toenails	No cutaneous lesions	Negative	Direct microscopy of nail scrapings (KOH)	1% lindane ointment
Taniguchi et al., 2017	2016	78/female	Japan	Not reported	Crusted scabies	Slightly thickened fingernails	Grayish erythema, scales, nodules on neck, trunk, arms and ankles	Not reported	Dermoscopy and microscopy	Topical 5% phenothrin with occlusive dressing
Zou et al., 2018	2018	55/male	China	non-Hodgkins lymphoma	Ordinary scabies	Thickened, yellowish, dystrophic fingernails/toenails	No cutaneous lesions	Not reported	Direct microscopy of nail scrapings (KOH)	Continuous occlusive dressing with crotamiton cream
Zaias et al., 2021	2021	83/male	USA	End-stage renal disease	Crusted scabies	Multiple small white superficial lesions on nail plate	No cutaneous lesions	Negative	Direct microscopy of nail scrapings (KOH)	Oral ivermectin plus topical permethrin under nail occlusion
Veraldi et al., 2025	2025	50/male	Italy	Healthy	Ordinary scabies	Second left toenail dystrophy	No typical scabies lesions	Not reported	Microscopic examination of nail/periungual scales	Topical 5% permethrin with occlusive dressing
Present case	2026	82/female	China	Chronic renal insufficiency and cerebral infarction	Nail-predominant scabies with concomitant onychomycosis	Four dystrophic, thickened, yellowish fingernails with periungual erythema, swelling, and hyperkeratotic scales	No cutaneous lesions	*Candida albicans* confirmed by fungal culture and MALDI-TOF MS	Direct microscopy revealed *Sarcoptes scabiei* mites/eggs and fungal hyphae simultaneously	Topical crotamiton under occlusive dressing combined with bifonazole cream

**Abbreviations**: KOH, potassium hydroxide; MALDI-TOF MS, matrix-assisted laser desorption/ionization time-of-flight mass spectrometry.

Our patient presented with localized nail dystrophy without typical cutaneous manifestations of scabies, making clinical diagnosis challenging. Unlike most previously reported cases [3], [8], in which concomitant fungal infection was either not specifically characterized or its clinical significance remained unclear, our patient demonstrated simultaneous fungal and parasitic involvement of the nail unit. MALDI-TOF MS identified the isolate as *C. albicans*, which represents an important but less common cause of onychomycosis [11]. Although onycholysis may facilitate secondary *Candida* involvement of the nail unit, its presence could not be reliably determined in our patient because it was not specifically documented at the initial examination. To the best of our knowledge, the coexistence of *C. albicans* onychomycosis and nail scabies has rarely been documented. The temporal relationship between *C. albicans* onychomycosis and nail scabies in our patient remains uncertain. One plausible explanation is that nail infestation by *S. scabiei* occurred first, with mite-associated hyperkeratosis and local inflammatory changes subsequently creating a microenvironment favorable for secondary *Candida* infection. Conversely, pre-existing onychomycosis might have altered the nail architecture and facilitated persistence of mites within the nail unit. Because both organisms were detected at the initial examination and no previous parasitological or mycological evaluation was available, the sequence of the two infections cannot be determined with certainty.

This case highlights the importance of considering nail scabies in the differential diagnosis of atypical nail disorders, even when onychomycosis appears to be the most likely clinical diagnosis. In patients presenting with unusual nail dystrophy, periungual inflammation, or poor response to antifungal therapy, direct microscopic examination of nail specimens should be performed to detect both fungal elements and parasitic infestation. A combined parasitological and mycological diagnostic approach may reduce misdiagnosis and facilitate appropriate treatment.

## CRediT authorship contribution statement

**Jie Zhou:** Writing – original draft, Methodology, Investigation, Funding acquisition, Formal analysis, Data curation, Conceptualization. **Nan Xu:** Writing – review & editing, Supervision, Investigation. **Min Chen:** Writing – review & editing, Writing – original draft, Supervision, Investigation, Funding acquisition, Formal analysis, Conceptualization.

## Ethics statement

As this was a study of case report, informed patient consent was required.

## Funding

This was funded by the subproject of the Pudong New Area National Comprehensive Reform Pilot Zone for Traditional Chinese Medicine Development (Grant No. PDZY-2024-0715) and the 10.13039/501100019978Medical Discipline Construction Program of Shanghai Pudong New Area Health Commission (the Key Disciplines Program PWZxk2025-01).

## Conflict of interest

None.

## Figures and Tables

**Figure:**
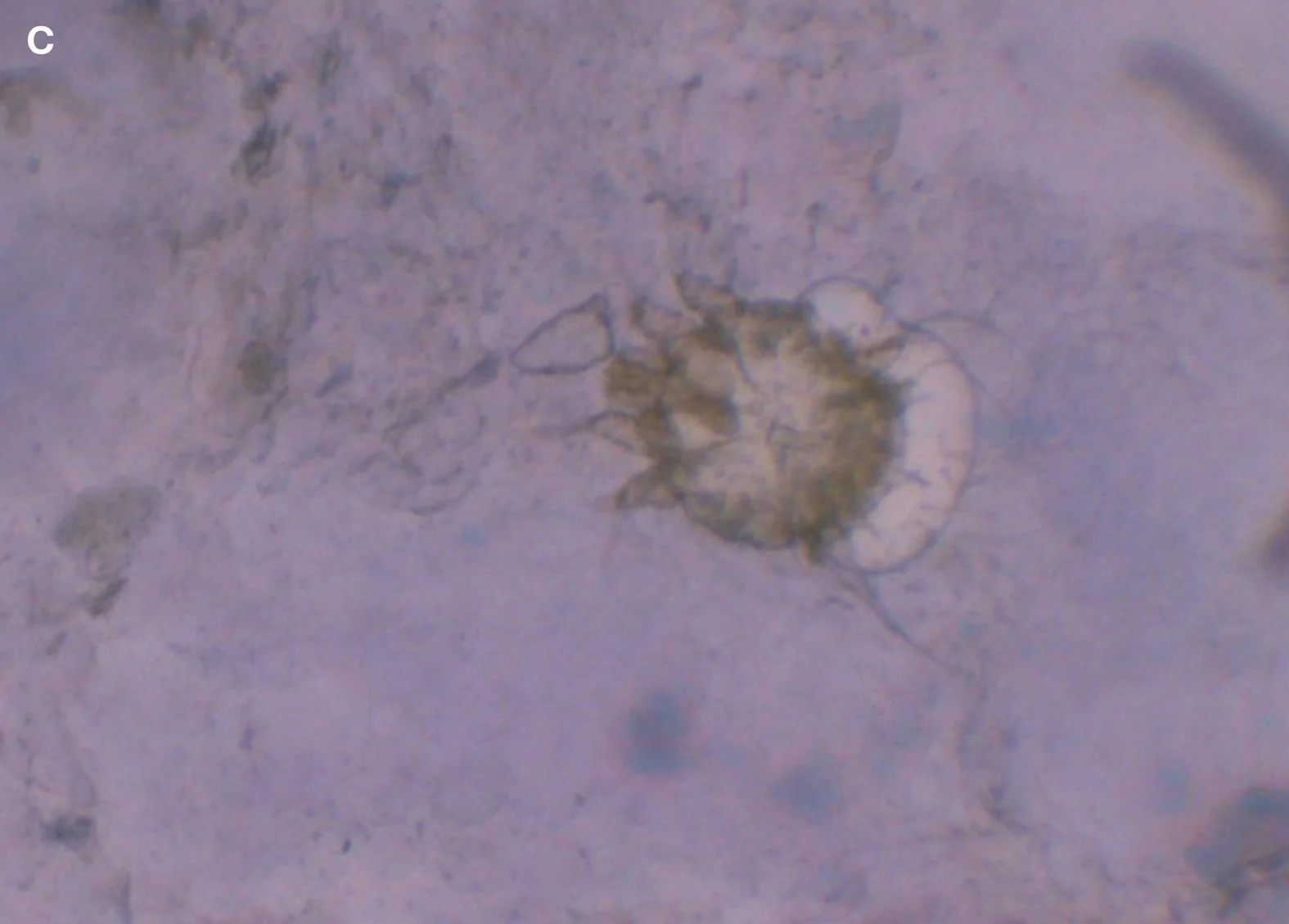
Figure S1. **Direct microscopic identification of *Sarcoptes scabiei* in nail specimens.** Direct microscopic examination of nail scrapings and subungual debris revealed a mite consistent with *Sarcoptes scabiei* within the hyperkeratotic material.

**Figure:**
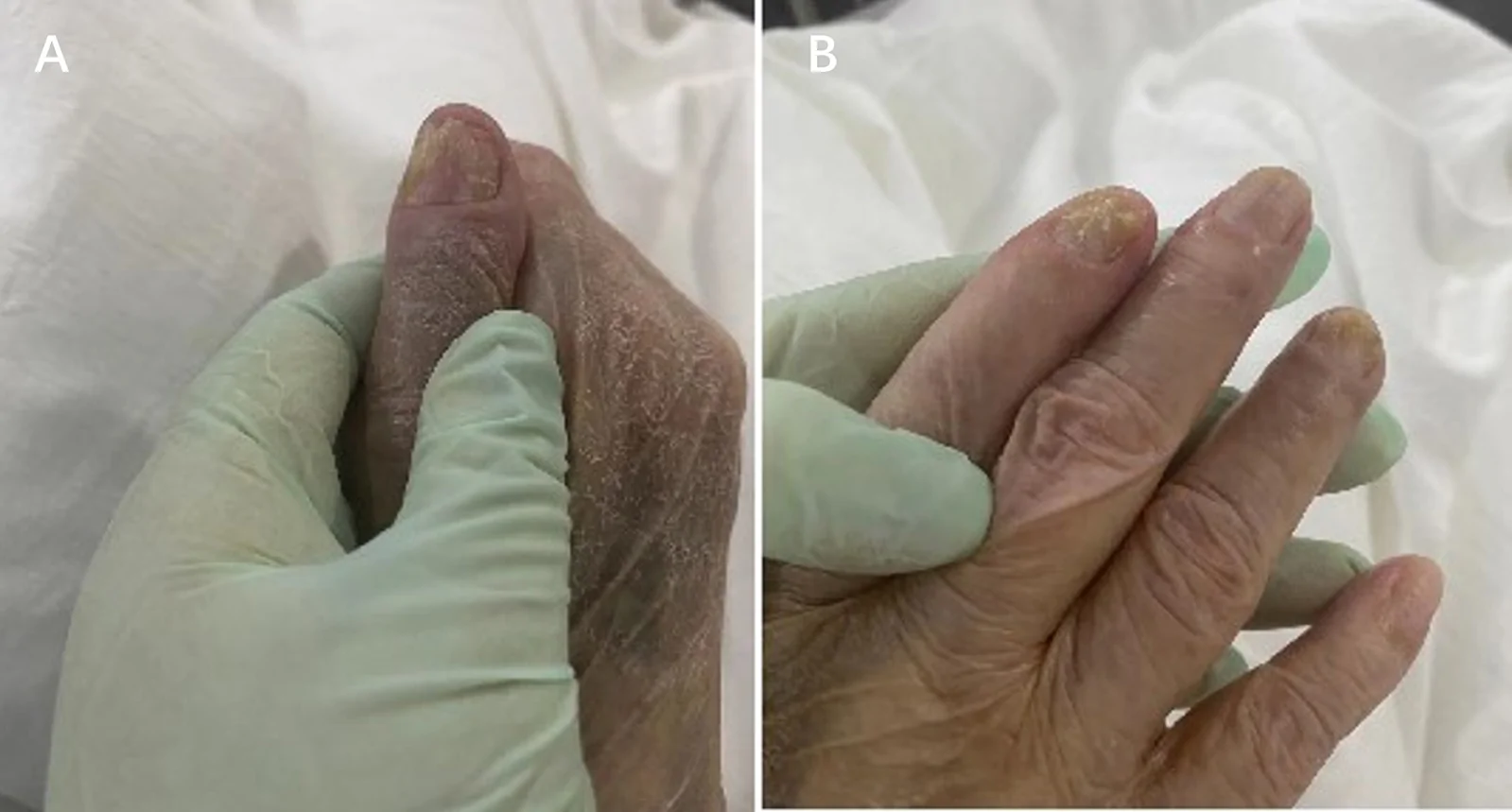
Figure S2. **Clinical improvement after combined antiparasitic and antifungal therapy.** (A, B) Significant improvement of nail dystrophy, periungual inflammation, and hyperkeratotic changes was observed after treatment with topical crotamiton under occlusive dressing combined with topical bifonazole.
